# The use of the international classification of functioning, disability and health (ICF) in indigenous healthcare: a systematic literature review

**DOI:** 10.1186/1475-9276-12-32

**Published:** 2013-05-16

**Authors:** Vanessa M Alford, Louisa J Remedios, Gillian R Webb, Shaun Ewen

**Affiliations:** 1 School of Physiotherapy, The University of Melbourne, Parkville, VIC, Australia; 2 Cente for Health and Society, Melbourne School of Population and Global Health, The University of Melbourne, Parkville, VIC, Australia

**Keywords:** ICF, Indigenous, Healthcare, Person centered, Bio-psychosocial, WHO, Health, Functioning, Review

## Abstract

**Introduction:**

The International Classification of Functioning, Disability and Health (ICF) was endorsed by the World Health Organisation (WHO) in 2001 to obtain a comprehensive perspective of health and functioning of individuals and groups. Health disparities exist between Indigenous and non-Indigenous Australians and there is a need to understand the health experiences of Indigenous communities from Indigenous Australian’s perspectives in order to develop and implement culturally appropriate and effective intervention strategies to improve Indigenous health. This systematic review examines the literature to identify the extent and context of use of the ICF in Indigenous healthcare, to provide the foundation on which to consider its potential use for understanding the health experiences of Indigenous communities from their perspective.

**Methods:**

The search was conducted between May and June 2012 of five scientific and medical electronic databases: MEDLINE, Web of Science, CINAHL, Academic Search Complete and PsychInfo and six Indigenous-specific databases: AIATSIS, APAIS-health, ATSI-health, health and society, MAIS-ATSIS and RURAL. Reference lists of included papers were also searched. Articles which applied the ICF within an Indigenous context were selected. Quantitative and qualitative data were extracted and analysed by two independent reviewers. Agreement was reached by consensus.

**Results:**

Five articles met the inclusion criteria however two of the articles were not exclusively in an Indigenous context. One article applied the ICF in the context of understanding the health experience and priorities of Indigenous people and a second study had a similar focus but used the revised version of the International Classification of Impairments, Disability and Handicap (ICIDH-2), the predecessor to the ICF. Four of the five papers involved Indigenous Australians, and one of the paper’s participants were Indigenous (First Nation) Canadians.

**Conclusion:**

Literature referring to the use of the ICF with Indigenous populations is limited. The ICF has the potential to help understand the health and functioning experience of Indigenous persons from their perspective. Further research is required to determine if the ICF is a culturally appropriate tool and whether it is able to capture the Indigenous health experience or whether modification of the framework is necessary for use with this population.

## Abstract (fr)

La Classification Internationale du Fonctionnement, de l’Incapacité et de la Santé (ICF) a été apprové par l’Organisation Mondiale de la Santé (OMS) en 2001 pour obtenir une perspective complète de la santé et du fonctionnement des individus et des groupes. Des disparités de santé existent entre les Australiens indigènes et non-indigènes et il y a un grand besoin de comprendre les états de santé des communautés indigènes vus de leur propre perspective afin de développer et mettre en application des stratégies d’intervention culturellement appropriées et efficaces pour améliorer leur santé. Cette révision systématique vérifie toute documentation pour identifier l’ampleur et le contexte de l’utilisation de l’ICF dans les soins des Indigènes permettant ainsi de considérer son utilisation potentielle pour comprendre les expériences de santé des communautés indigènes vues de leur propre perspective.

**Méthodes**

La recherche a été accomplie entre mai et novembre 2012 à partir de cinq bases de données électroniques scientifiques et médicales: MEDLINE, Web of Science, CINAHL, Academic Search Complete, PsychInfo et six bases de données spécifiquement indigènes: AIATSIS, APAIS-health, ATSI-health, health and society, MAIS-ATSIS et RURAL. Les listes de références des documents inclus ont été également examinées. Les articles qui s’appliquaient à l’ICF dans le contexte indigène ont été sélectionnés. Des données quantitatives et qualitatives ont été extraites et analysées par deux critiques indépendants et les désaccords ont été résolus par consensus.

**Résultats**

Cinq articles ont bien répondu aux critères d’inclusion, bien que deux des articles n’aient pas fait exclusivement partie d’un contexte indigène. L’un des articles a appliqué l’ICF pour comprendre les expériences et les priorités de santé des Indigènes et une deuxième étude a fait de même mais a employé une nouvelle version de la Classification Internationale de l’ Infirmité, l’Incapacité et le Handicap (ICIDH-2), le prédécesseur de l’ICF. Tous les documents concernaient les Indigènes australiens à l’exception d’un dont les participants étaient des Indigènes (Premières Nations) canadiens.

La documentation se rapportant à l’utilisation de l’ICF avec des populations indigènes est limitée. L’ICF a le potentiel d’aider à comprendre les expériences de santé et le fonctionnement des Indigènes vus de leur propre perspective. Davantage de recherche est essentielle pour déterminer si l’ICF est culturellement appropriée et peut capturer les expériences de santé des Indigènes ou si une modification de cadre est nécessaire pour son utilisation avec cette population.

## Introduction

### The international classification of functioning, disability and health

**Figure 1:**
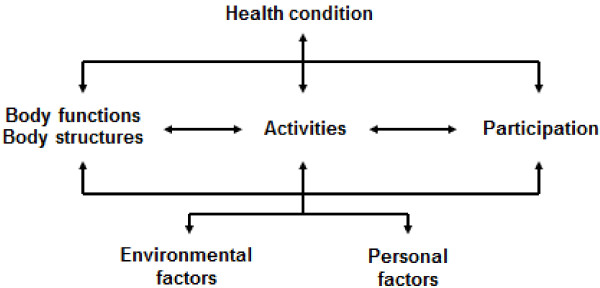
The international classification of functioning, disability and health WHO, (2001).

The International Classification of Functioning, Disability and Health (ICF) (WHO, 2001) (Figure 1) is a framework premised on the bio-psychosocial model that was endorsed by the World Health Organisation (WHO) in 2001 to give a comprehensive perspective of health and functioning at both individual and population levels [1]. The ICF was a successor to the International Classification of Impairments, Disabilities and Handicaps (ICIDH), (WHO, 1980) which was developed to capture the overall health status of populations but which focused more on disease and failed to capture the impact of the social and physical environment on functioning [2]. In 1993, in response to the need for a model which considered the influence of contextual factors on one’s health experience at individual and population levels, a revision of the ICIDH began. Nearly ten years of collaboration between government and non-government organisations, including groups representing people with disabilities resulted in the revised version of the ICIDH (ICIDH-2). This was developed in multiple languages, renamed the ICF and officially endorsed in May 2001.

The ICF uses more positive terminology than its predecessor and shifts the focus from the cause of the disease to its impact on functioning [3]. It looks beyond the physical impairments of the individual, recognising that functioning, disability and quality of life are not only the consequence of biological dysfunction but are a result of the interaction between the health condition, biomedical factors and the social, personal and environmental factors, including the performance of activities and participation in life situations [4]. Exploring contextual factors allows for a greater understanding of the whole person and recognises the variation in health experiences depending on an individual’s circumstances. The ICF has been used to inform assessment questions and to conceptualise the experiences and needs of people with a wide range of health conditions [3,5-7]. By encouraging a comprehensive understanding of a person’s health and functioning experience, including their ability to participate in activities and life situations and the overall physical, social and attitudinal environment in which the person lives, the ICF makes it is possible to identify the needs of the person beyond those described in the diagnosis [8]. Furthermore, the functional goals of the individual may be better understood and appropriate intervention options provided which support these goals [3].

### Australian indigenous health

The health of Indigenous Australians^a^ remains well below that of other Australians with disparities between the two groups widening [9]. It is therefore imperative that current health practices used in Indigenous healthcare are explored and models that are more congruent with Indigenous health views and values are evaluated for potential use in understanding the health experience of the Indigenous population.

From an Indigenous perspective, health extends beyond the traditional biomedical model to a broader concept which implies “a more holistic, community focused and spiritual understanding of human health” [10] influenced strongly by contextual factors, including historical, social and cultural factors and connection to land and country [11]. While Indigenous Australians have demonstrated resilience in surviving the challenges they have been faced with, “colonisation and subsequent assimilation policies have undermined Indigenous culture and spiritual identity” [12], destroying their autonomy and self-determination [13] and depriving them of community control and their ability to participate fully in society which continues to have a devastating impact on Indigenous health [14].

*“Our identity as human beings remains tied to our land, to our cultural practices, our systems of authority and social control…. Destroy this relationship and you damage…individual human beings and their health”* (Anderson 1996) in [15].

Despite the widely acknowledged complexity of the determinants of Indigenous health there is evidence that many approaches to Indigenous healthcare still primarily employ the biomedical model [16]. This approach focuses predominantly on the physical ailments of the disease and ignores the social and cultural influences on Indigenous health. There is a need to look beyond this model, to a bio-psychosocial approach which can help to better understand the Indigenous health experience and the needs of Indigenous communities [15-18]. Because the ICF recognises that multiple influences contribute to a person’s health experience [19], including psychological, social and environmental aspects of everyday life, it has the potential to be applied in the indigenous healthcare context.

### Patient-centered approach

There is also growing recognition of the importance of adopting a patient-centered approach in Indigenous healthcare [20,21] to acquire a broader understanding of the Indigenous health experience from the patient perspective, and so more effectively address the health needs of Indigenous communities [22]. A patient-centered approach is a collaborative process which actively involves eliciting the individual’s narrative in the consultation process [23]. This approach, which has been shown to increase the impact of primary health care in Indigenous communities [24] was advocated by Enid Balint over forty years ago to better understand the patient and their needs rather than merely fitting them into predetermined criteria based on their illness [25]. In the 1970’s George Engel also recognised the need to focus not only on the illness but on the patient, including the psychological and social influences on one’s health and integrated a patient-centered approach into a bio-psycho-social model of health [26].

In Indigenous communities, seeking information is a two-way process where both parties contribute information [22]. However, inappropriate and interrogational interviewing of Indigenous patients by mostly non-Indigenous health professionals and miscommunication regarding important health issues due to “dominance of the biomedical model,….lack of control by the patient (and) lack of shared knowledge and understanding” have been reported as significant barriers to effective healthcare [22,27] and may offer some explanation as to why many current interventions in Indigenous health care are not based on Indigenous perceptions and needs [19]. Adopting a patient-centered approach, which lightens the directive demeanour and notion of the practitioner as the power figure and shifts the focus towards patient autonomy, may help to reduce these barriers by “increasing Indigenous involvement in overall management processes” [22] and improving communication between non-Indigenous health professionals and their Indigenous patients. Understanding the patient’s experience of health from their perspective is likely to result in more effective, culturally appropriate interventions based on individual needs [28,29].

The ICF has been reported to be a useful tool for understanding health and functioning from the patient perspective, including the influence of contextual factors [3,5-7,30,31] and for providing a comprehensive analysis of a person’s experiences and needs [3,5-7,32,33]. It has been applied across various health disciplines [34] and in countries in all six WHO regions^b^[6], however it is not known whether the ICF has been used in an Indigenous context.

### Previous reviews of the ICF

Previous reviews of the ICF have found an increase in publications reporting on its use in healthcare since its endorsement in 2001 [34,35]. A review by Jelsma (2009) found that the ICF has been used across disciplines, health conditions, sectors and settings and that it has made an impact on data collection and analysis of people with disabilities. Cerniauskaite et al. (2011) performed a review of the literature on the ICF from 2001 to 2009 and also found a significant increase in the quantity of globally published literature during this time with one third of identified articles published in 2008 and 2009. It was found that the ICF has been used in both clinical and non-clinical contexts, including legislation, labour, education and policy development [35]. Fayed et al. (2011) performed a systematic review on the use of ICF linking rules for linking health and health-related information to the ICF and found evidence that this method was used for describing and comparing information from outcome measures, results from qualitative research and clinical patient reports [36]. However none of the reviews made reference to Indigenous healthcare or other minority populations so it is not known whether the ICF has been applied in an Indigenous context.

### Review objective

The objective of this review is to identify whether the ICF has been used in Indigenous healthcare and in which context, to provide the foundation on which to consider its potential use for understanding the health and functioning experiences of Indigenous communities from their perspective.

## Methods

### Data sources

A literature search was conducted using five electronic databases: MEDLINE, Web of Science, CINAHL, Academic Search Complete and PsychInfo as well as the following Indigenous databases: AIATSIS, APAIS-health, ATSI-health, health and society, MAIS-ATSIS and RURAL between June and August 2012.

The following search terms were searched for in the title, abstract and keywords: “International Classification of Functioning, Disability and Health” OR “International Classification of Functioning” AND Indigenous OR Aborigin* OR “First Nation” OR Koori OR “Torres Strait Island*” OR “Oceanic ancestry group” OR Koori OR Yolngu OR “First Nation” OR Inuit OR “Native Australia*”. Advice was sought from two experts in Indigenous health on which search terms to use. Truncation marks were used to increase the depth of the search. The search was set from 2000, the year before the ICF was endorsed to 2012. The search was restricted to papers in English for practical purposes. Reference lists of retrieved studies were also searched to identify additional studies.

### Study selection

**Figure 2:**
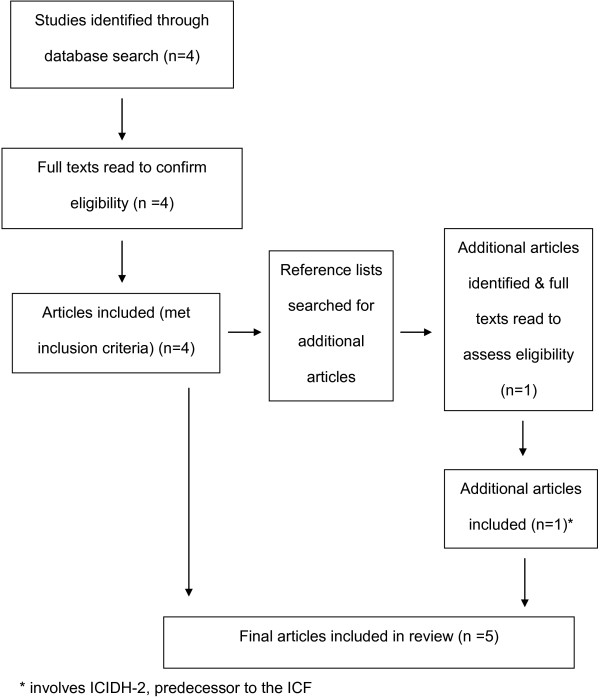
Selection process for inclusion of studies in the systematic review.

The selection process for inclusion of studies in our review is illustrated in Figure 2. Full texts of the articles retrieved from the database search were read and selected based on the following inclusion criteria: 1) ICF applied or referred to in healthcare 2) sample involved Indigenous persons. A second reviewer (LR) independently screened the articles to confirm study selection. References lists of selected articles were then read to identify any additional relevant articles and full texts of these articles were read to determine their eligibility in the review.

### Data extraction

A data extraction spread sheet was developed by the first author (VA) and used to capture quantitative and qualitative data. Quantitative variables included the year of publication, whether the sample was exclusively Indigenous, the origin of the Indigenous group, and whether the ICF was used to understand the person-perspective. Narrative data was entered verbatim into the spread sheet under the categories ‘Aim of study/article’, ‘Context of ICF use’, ‘Reported conclusions regarding use of the ICF’ and ‘ICF limitations reported’. The data was extracted independently by the primary author (VA) and a second reviewer (LR). High levels of agreement existed between the two reviewers with consensus reached in discussion over some of the language choices.

## Results

Four papers were identified from the database search which met the inclusion criteria [37-40]. An additional study was identified through the reference list search of selected articles which was also eligible for inclusion [41].

A summary of the included articles is presented in Table 1 and the data extracted regarding use of the ICF is summarised in Table 2. Five articles met the inclusion criteria of which three were exclusively in an Indigenous context [37,38,41]. Of these three papers, two were reports of studies which aimed to understand the Indigenous health experience and priorities of Indigenous people from the person perspective [38,41] however one of these studies used the ICIDH-2, the predecessor to the ICF [41].

**Table 1:** Summary of articles included in the review

** Author**	**Year**	**Aim of study/article**	**Indigenous origin**	**Exclusively Indigenous**
Beaudin	2010	To examine the similarities and differences in worldview of health and healing between Aboriginal Canadians and ’Western Europeans’ as expressed in the ICF, and to explore the extent to which these can be ‘converged’ to create an inclusive health policy and service for Aboriginal Canadians.	Canadian	Yes
Senior	2000	To explore the extent to which the ICDH-2* captures the health experience of Indigenous Australians with specific attention to participation preferences. It further explored the perception of disability in two Indigenous communities.	Australian	Yes
Lindeman	2006	To investigate the assessment of client needs in remote Home and Community Care services and report on workshops aimed at improving community care assessment skills of staff.	Australian	Yes
McCormack	2011	To examine the association between communication impairment and children’s activities and participation.	Australian	No - 2.8 % of sample
Dew	2012	To report on the current therapy service delivery model in rural and remote communities in New South wales, Australia.	Australian	No

*The ICIDH-2 is the predecessor to the ICF and includes the same components as the ICF.

**Table 2:** Context of ICF use, conclusions and limitations reported

** Author**	**Context of ICF use**	**ICF used to understand person-perspective?**	**Conclusions reported regarding ICF**	**Limitations of ICF reported**
Beaudin	To express worldviews of health and healing of Aboriginal Canadians and explore how culture influences the ICF components.	Yes	Use of the ICF should incorporate attention to culture under the Personal factors component. Awareness of how cultural factors influence other elements of the ICF is needed.	None
Senior	Used the ICIDH-2 to understand health experiences of people in two Indigenous communities.	Yes	Flexible tool which covers most life experiences of Indigenous people. Concepts of activity and participation translated well for individuals’ daily experience.	Spiritual significance of traditional practices, shame, isolation and loneliness not captured.
Lindeman	Workshops for HACC assessors included a discussion around the ICF as a framework for assessment.	No	Appropriate, relevant, acceptable and easy to use.	None
McCormack	Used the ICF-CY as a reference for outcome measures.	No	None	None
Dew	Briefly mentions activities component of ICF.	No	None	None

The study by Beaudin (2010) was an ethnographic study on the perspective of Aboriginal Canadians^c^, contrasting their beliefs and worldview of health and healing with the Western view and was the only piece of literature identified which did not involve Indigenous Australians. The findings illustrated the significance of culture on the health experience of Aboriginal Canadians. It was suggested that use of the ICF should incorporate attention to culture under the personal factors component to understand its influence on the elements of the ICF. Used in this context Beaudin suggested that the ICF may be useful to help understand the experience Aboriginal people and so assist in informing culturally appropriate changes to existing health policy and services for Aboriginal Canadians.

The other study which incorporated a person-centered approach^d^ to understand people’s health and functioning was identified in the reference list search [41]. The aim of this study was to examine individual and community perceptions of disability in a remote and an urban Indigenous community using the ICIDH-2, with specific attention to participation preferences, to determine its relevance in an Indigenous context. Because the ICIDH-2 is an updated version of the ICIDH and was soon after renamed and endorsed as the ICF, the decision was made to include it in our review. The study found that participation in life activities and involvement in community was more influential on health than biomedical markers of disease and was significantly influenced by contextual factors such as social support and the physical environment, all of which were captured in the ICIDH-2. Shame, isolation and loneliness associated with being treated differently also directly influenced community participation and were reported to be a cause of ill health however the ICIDH-2 was not able to capture these factors.

The third article in our review, by Lindeman & Newman (2006), included a discussion on the ICF as a conceptual framework for conducting needs assessment, in workshops aimed at improving community care assessment skills of staff in remote Indigenous communities as part of the Home and Community Care program. Although the ICF was not implemented in a practical setting, workshop participants believed it would be “appropriate, relevant, acceptable and easy to use” [37].

The remaining two articles were not exclusively in an Indigenous context [39,40]. McCormack (2011) used the ICF as a framework as part of a longitudinal study which investigated activity and participation in children with communication impairment however a nationally representative study sample meant that only 2.8% of the sample was Indigenous and the author did not report on the usefulness or limitations of the ICF with this population group as this was not the focus of the study.

Dew (2012) examined therapy service delivery models to non-Indigenous and Indigenous people living in outer regional and remote areas of Australia, but only once mentioned the ‘activities’ component of the ICF in stating that Indigenous people are twice as likely to experience activity restriction compared to non-Indigenous people.

## Discussion

Our review provides evidence of the limited use of the ICF in Indigenous healthcare since its endorsement in 2001. Aside from the study by Beaudin (2010) it appears that no studies have focussed on using the model to understand Indigenous health and functioning. These findings are consistent with our knowledge and a discussion with experts in Indigenous health.

The findings of the study by Senior (2000) depict the importance of moving beyond the traditional biomedical framework and adopting a person-centered approach using a bio-psychosocial model, in order to better understand the Indigenous health experience, including their functional and participation priorities. For the communities studied, perception of good health did not necessarily mean “freedom from disease and unrestricted bodily functioning” [41] and people were not worried about their health if they could “keep going as usual” [41]. The diverse perceptions of “normal participation” at different life stages was also recognised, which stresses the importance of understanding participation preferences and priorities from the person-perspective in order to meet individual needs. This becomes even more essential when the diversity of Indigenous communities is considered [41]. Overall the ICIDH-2 was found to be a flexible tool which covers most life experiences of Indigenous people including their needs and the contextual influences on participation, which advocates the suitability of the ICF model to Indigenous healthcare. The fact that the framework was unable to capture the spiritual significance of traditional practices and the consequence of shame, isolation and loneliness is consistent with results of other studies which reported that the ICF does not incorporate the context of emotions [5,42], sense of self or self-satisfaction [43]. Further research should consider whether these factors can in fact be captured within the personal factors component of the ICF or whether modification to the framework may be necessary.

Findings from Beaudin (2010) illustrate the significance of culture on the health experience of Aboriginal Canadians, which reinforces the need for culturally appropriate models in Indigenous healthcare and the need for healthcare professionals to understand how their own cultural values operate against those of Indigenous patients [16,20,44,45]. Inappropriate attitudes and lack of cultural competence of healthcare practitioners have been documented as barriers to healthcare for Indigenous Australians [46] and although not deliberate, the ignorance displayed towards other cultural values by health professionals and the presumption that their culture is the norm to which others should conform is a subconscious act of racism [22]. The importance of acknowledging cultural influences on illness across all population groups has been understood for many years [47] and is fundamental for Indigenous people, for whom spiritual and cultural aspects of health are just as important, if not more important, than the physical.

As stated by Garcia (2002) *“Acknowledging and incorporating cultural beliefs about health and illness….contributes to successful healthcare”* cited in [22].

As emphasised by Beaudin (2010), the importance of collecting cultural knowledge and understanding how this may influence the other elements in the ICF should be taught to health professionals as a way of building their cultural competence in Indigenous healthcare.

### The potential of the ICF for understanding the Indigenous health experience

Despite potential barriers to its use, findings of the studies by Senior (2000) and Beaudin (2010) suggest the ICF may be suitable to address the need for a unified bio-psychosocial framework in Indigenous healthcare. The authors suggest the ICF may facilitate a better understanding of the health and functioning experience of Indigenous communities from the person perspective, including the issues important to them and the influence of cultural and other contextual factors. The ICF has been used internationally [6] and across a range of conditions to understand health and functioning from the person perspective [3,6,30,31,42,43]. It was developed to be applicable to cultures worldwide [48] and has been found to capture some Indigenous cultural beliefs and values [38]. Furthermore, the ICF is reported to be a “unified, international and standardised language” [48] meaning that understandings can be shared across disciplines [49] and between health professionals and patients. We therefore propose that there is potential to use the ICF to communicate and translate the Indigenous experience into a framework to help conventionally trained health professionals better understand Indigenous experiences and values. This may not only improve communication between practitioners and patients but it may lead to more appropriate and effective intervention strategies and enhance the healthcare experience of Indigenous people [29]. Understanding the lived experience from the Indigenous perspective may also help inform policy and service delivery to better meet the needs of Indigenous communities.

The determinants and antecedents of Indigenous health are complex and future research is required to confirm whether the ICF is suitable for use with this population or whether modifications to the framework are necessary to ensure all determinants of Indigenous health, including family, community and spiritual practices are integrated in the model to capture the lived experience of Indigenous people at both an individual and community level. If the ICF does prove to be a suitable model for use in this context, it could also potentially be used with other underrepresented minorities.

### Limitations of the review

As with any review there is the potential of omitting relevant articles and unpublished material and the findings of this study are limited to literature in the English language. However steps were taken to exhaust the literature during the period of data collection, including searching Indigenous-specific databases and hand searching reference lists. Despite the potential omission of relevant articles, our review provides a respectable indication of the limited use of the ICF in Indigenous healthcare.

## Conclusions

To our knowledge, evidence surrounding the use of the ICF with Indigenous populations is scarce. Since its endorsement in 2001, only one study involving the ICF has investigated Indigenous beliefs and experiences of health from the person perspective [38] and this was in the Canadian context. In addition, Senior (2000) conducted a study using the ICIDH-2, the predecessor to the ICF and reported on its potential use in better understanding the Indigenous health experience.

The health and functioning experience of Indigenous communities and their perception of health and illness, including aetiology of disease, participation preferences and the influence of contextual factors on their health differ in comparison to mainstream society. There is also considerable variation in perceptions and values of health within Indigenous communities [41] which can be attributed to the diversity of Indigenous communities. Therefore acknowledging and understanding Indigenous perspectives is vital in order to implement appropriate and effective management strategies for people with health conditions [50]. Integrating a person-centered approach into a model which doesn’t neglect the socio-cultural influences on health is paramount and supports the development of a deeper understanding of the health and functioning experience of Indigenous communities from the person perspective. The ICF framework, which pays more holistic attention to the individual, including their participation preferences and the contextual factors which impact health and functioning, has potential to be used in this context. Further research is required to determine the relevance of the ICF components to Indigenous health and whether the model is sensitive enough to capture the complexity of Indigenous health. Potential barriers to the use of the ICF framework in Indigenous healthcare have been identified so it may be that it needs to be modified for use with this population. If the ICF does prove to be suitable for use in Indigenous health, it has the potential to provide a better healthcare experience for Indigenous people and an avenue to reducing the disparities in healthcare accessed between Indigenous and non-Indigenous Australians.

## Endnotes

^a^In Australia the term Indigenous includes Aboriginal and Torres Strait Islander peoples and represents a diverse range of communities. Indigenous Australians are the original inhabitants of the country and currently comprise approximately 3.0% of Australia’s population.

^b^The WHO geographical regions are Africa, Americas, Eastern Mediterranean, Europe, South-East Asia and Western Pacific. (WHO: Definition of region groupings. Retrieved from http://www.who.int/healthinfo/global_burden_disease/definition_regions/en/index.html).

^c^Beaudin (2010) uses the term ‘Aboriginal’ to refer to the Indigenous people of Canada. The communities in which the research was carried out in this study were First Nation and Metis communities.

^d^In this review we use the terms person-centered and patient-centered interchangeably depending on the context which we are referring to.

## Abbreviations

AIATSIS: Indigenous studies bibliography; APAIS-health: Australian public affairs information service – health; ATSI health: Aboriginal and Torres Strait Islander health bibliography; Health and Society: Health and Society Database; ICF: International classification of functioning, health and disability; ICIDH: International classification of impairment, disability and handicap; MAIS-ATSIS: Multicultural Australia and immigration studies – Aboriginal and Torres Strait Islander subset; RURAL: Rural and remote health databases; WHO: World health organisation.

## Competing interests

The authors declare that they have no competing interests.

## Authors’ contributions

VA developed the study design, carried out the literature review and analysis and drafted the manuscript. LR independently reviewed the identified articles to confirm their eligibility for inclusion in the review, analysed their content and contributed to drafting and critically revising the manuscript. SE contributed to the drafting and critical revision of the manuscript for important intellectual content. All authors contributed to intellectual property and approved the final manuscript.

## Authors’ information

VA is currently a PhD student at the School of Physiotherapy, The University of Melbourne. The focus of her PhD is on considering the suitability of the ICF to better understand the Indigenous experience of health and functioning from the person perspective and as a common framework in health professional education.

Associate Professor SE is the inaugural Associate Dean (Indigenous Development) Faculty of Medicine, Dentistry and Health Science, at The University of Melbourne. His research interests and expertise are in workforce development, Indigenous health, and medical education.

Doctor LR is a senior lecturer and the coordinator of teaching and learning in the Physiotherapy department, The University for Melbourne. Her research interests include the use of sociocultural frameworks for interpreting health and wellness experiences and cultural literacy in the healthcare domain.

Associate Professor GW has held a variety of academic positions in physiotherapy education over many years. Her research interests include global health, indigenous health and cultural influences on health and wellbeing. She is president of the International Society of Physiotherapy Educators.
